# 3-Hydroxyphenylacetic Acid: A Blood Pressure-Reducing Flavonoid Metabolite

**DOI:** 10.3390/nu14020328

**Published:** 2022-01-13

**Authors:** Patrícia Dias, Jana Pourová, Marie Vopršalová, Iveta Nejmanová, Přemysl Mladěnka

**Affiliations:** 1 Department of Pharmacology and Toxicology, Faculty of Pharmacy, Charles University, Akademika Heyrovskeho 1203, 500 05 Hradec Kralove, Czech Republic; diasp@faf.cuni.cz (P.D.); voprsalova@faf.cuni.cz (M.V.); mladenkap@faf.cuni.cz (P.M.); 2 Department of Biological and Medical Sciences, Faculty of Pharmacy, Charles University, Akademika Heyrovskeho 1203, 500 05 Hradec Kralove, Czech Republic; najmanoi@faf.cuni.cz

**Keywords:** flavonoids, gut microbiota, metabolite, 3-hydroxyphenylacetic acid, blood pressure, vasorelaxation, coronary, artery, rat, pig

## Abstract

Regular intake of polyphenol-rich food has been associated with a wide variety of beneficial health effects, including the prevention of cardiovascular diseases. However, the parent flavonoids have mostly low bioavailability and, hence, their metabolites have been hypothesized to be bioactive. One of these metabolites, 3-hydroxyphenylacetic acid (3-HPAA), formed by the gut microbiota, was previously reported to exert vasorelaxant effects ex vivo. The aim of this study was to shed more light on this effect in vivo, and to elucidate the mechanism of action. 3-HPAA gave rise to a dose-dependent decrease in arterial blood pressure when administered i.v. both as a bolus and infusion to spontaneously hypertensive rats. In contrast, no significant changes in heart rate were observed. In ex vivo experiments, where porcine hearts from a slaughterhouse were used to decrease the need for laboratory animals, 3-HPAA relaxed precontracted porcine coronary artery segments via a mechanism partially dependent on endothelium integrity. This relaxation was significantly impaired after endothelial nitric oxide synthase inhibition. In contrast, the blockade of SKCa or IKCa channels, or muscarinic receptors, did not affect 3-HPAA relaxation. Similarly, no effects of 3-HPAA on cyclooxygenase nor L-type calcium channels were observed. Thus, 3-HPAA decreases blood pressure in vivo via vessel relaxation, and this mechanism might be based on the release of nitric oxide by the endothelial layer.

## 1. Introduction

Cardiovascular disease is the leading cause of death worldwide, with an estimated number of deaths approaching 18 million per year. Coronary artery disease and stroke contribute to approximately 85% of these fatal cardiovascular events [1]. The global mortality and morbidity have prompted the implementation of guidelines for prevention of cardiovascular risks [2]. The detection of persistent high arterial blood pressure with subsequent treatment is among the main strategies [3,4]. Indeed, hypertension is classified as a major risk factor for cardiovascular diseases. It is mostly caused by increased systemic vascular resistance, and this is often related to various structural and functional changes in the vasculature, which disrupt vascular homeostasis. In particular, the tunica intima of blood vessels, formed by a single layer of endothelial cells, is dysfunctional. In addition, the vascular smooth muscle cells often appear more contracted and less responsive to endothelium-derived relaxing factors [5].

**Figure 1:**
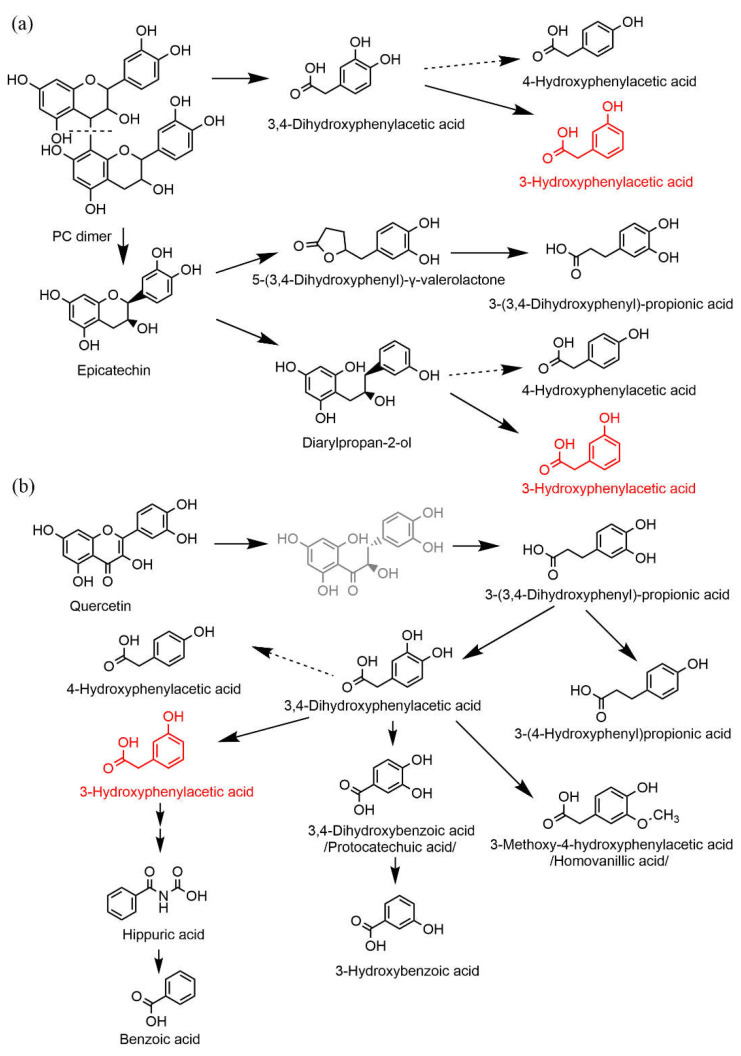
Simplified schemes of proposed pathways for microbial degradation of different flavonoids in humans: flavanols (**a**) and flavonol quercetin (**b**). PC: procyanidin. 3-hydroxyphenylacetic acid (3-HPAA) is depicted in red; the structures in black are metabolites that were detected, whereas structures in grey represent hypothetical intermediates. Data were obtained from the following references: [16,17,20,21,22,23].

Several studies have provided evidence of the protective effects of a polyphenol-rich diet against cardiovascular diseases. Indeed, a diet rich in fruits, vegetables, nuts, chocolate or tea, which are rich sources of flavonoids, has been associated with a reduction in the risk of cardiovascular diseases [6]. In addition to antioxidant, anti-inflammatory, antiplatelet, and antimicrobial properties, flavonoids also possess significant vasodilatory properties [7,8]. However, flavonoid intake worldwide is highly variable and, once ingested, these compounds interact in a complex manner with the gastrointestinal tract. Briefly, flavonoids occur in plants mostly in the form of glycosides. After administration in the form of diet, they are hydrolyzed during digestion and this allows the release of the aglycone form in the small intestine. Aglycones, in turn, can undergo metabolism in the epithelium of the small intestine and later in the liver. This results in a very poor bioavailability of parent flavonoids, which was repeatedly confirmed [9,10]. However, the unabsorbed fraction passes from the small intestine into the distal gut, where it is cleaved by the colonic microbiota leading to the production of small phenolic compounds, including phenylpropionic, phenylacetic, and benzoic acids, and hydroxybenzenes, which are subsequently absorbed [9]. It is noteworthy that over the last few decades, it has become clear that the protective effects of dietary flavonoids are attributed to their metabolites rather than their parent forms [10,11,12]. Notwithstanding, Booth and colleagues documented even in the early 1950s the presence of various flavonoid metabolites, namely 3,4-dihydroxyphenylacetic (DHPA), 3-hydroxyphenylacetic (3-HPAA) and homovanillic acids in the urine of different animal species after oral ingestion of quercetin [13]; research on the biological effect of small phenolic metabolites of flavonoids is much more recent. Specifically, in recent years, research on the metabolic fate of flavonoids as well as studies reporting bioactivities of their microbe-derived metabolites have become a hot and intensively investigated topic in the field. Our research group has shown that several microbial metabolites, including DHPA, 4-methylcatechol and 3-(3-hydroxyphenyl) propionic acid, relax rat aorta in likely physiologically achievable concentrations [14,15]. To our best knowledge, 3-HPAA is one of the least explored metabolites. It was documented to be a metabolite of a number of phenolic compounds including different classes of flavonoids such as flavonols (quercetin with its glycoside rutin, kaempferol), isoflavones, and flavanols (both oligomeric procyanidins and their monomeric units, catechin and epicatechin) (Figure 1) [16,17,18,19,20]. 

As 3-HPAA relaxes smooth muscle cells ex vivo [14], this study aimed to test if this metabolite is able to cause a decrease in arterial blood pressure in vivo and to detect its mechanism of action in a series of mechanistic experiments. 

## 2. Materials and Methods

### 2.1. Animals

The in vivo experiments were carried out on spontaneously hypertensive rats (SHR) obtained from the Czech Academy of Sciences, Prague, Czech Republic. The animals were bred in the animal facility of the Faculty of Pharmacy and maintained at a constant temperature of 23–25 °C with a 12 h dark/light cycle. Rats were provided a standard diet and tap water ad libitum. The study (reg. No. 4937_2019-9) was approved by the Ministry of Education, Youth and Sports, conforming to The Guide for the Care and Use of Laboratory Animals published by the US National Institutes of Health (8th edition, revised 2011, ISBN-13: 978-0-309-15400-0).

### 2.2. Chemicals

Pentobarbital, sodium nitroprusside, dimethyl sulfoxide (DMSO), L-Nω-nitro arginine methyl ester (L-NAME), atropine, TRAM-34/1-[(2-chlorophenyl)diphenylmethyl]-1H-pyrazole, indomethacin, nifedipine, bradykinin, KCl, CaCl_2_, and UCL-1684/6,12,19,20,25,26-hexahydro-5,27:13,18:21,24-trietheno-11,7-metheno-7H-dibenzo[b,m][1,5,12,16]tetraazacyclotricosine-5,13-diium ditrifluoroacetate hydrate were purchased from Sigma-Aldrich (Steinheim, Germany). Bay K8644 was obtained from Axon Medchem BV (Groningen, The Netherlands). The tested metabolite 3-HPAA was purchased from Toronto Research Chemicals (Toronto, ON, Canada). NaCl, NaHCO_3_, and D-glucose were provided by PENTA s.r.o (Prague, Czech Republic). MgSO_4_·7H_2_O was purchased from Erba Lachema s.r.o. (Brno, Czech Republic), and KH_2_PO_4_ from Dr. Kulich Pharma s.r.o. (Hradec Králové, Czech Republic). Saline was purchased from Baxter Czech spol. s.r.o. (Prague, Czech Republic) and heparin from Zentiva (Prague, Czech Republic).

Sodium nitroprusside, L-NAME, and atropine were dissolved in distilled water. Stock solutions of TRAM-34 (1 mM) and UCL-1684 (100 µM) were prepared as water solutions supplemented with 20% and 10% DMSO, respectively. A stock solution of indomethacin (1 mM) was also prepared in 10% DMSO/water, and Bay K8644 (10 mM) and nifedipine (10 mM) were dissolved in pure DMSO. The final concentration of DMSO in the tissue chamber was always ≤0.2%. The Krebs solution contains the following (mM): NaCl 119, KCl 4.7, CaCl_2_ 1.25, KH_2_PO_4_ 1.18, MgSO4·7H_2_O 1.17, NaHCO_3_ 25, and D-glucose 11. 

### 2.3. In Vivo Experiments

**Figure 2:**
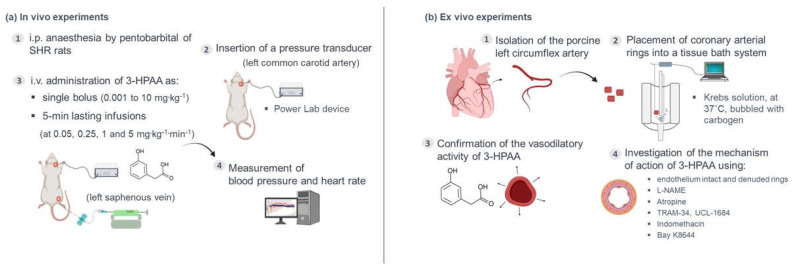
Graphical scheme of the performed experiments. The first panel (**a**) depicts the in vivo experiments performed: (1) spontaneously hypertensive rats (SHR) were anesthetized i.p. by pentobarbital (50 mg·kg^−1^); (2) a pressure transducer was linked to the left common carotid artery; (3) in one set of experiments, 3-hydroxyphenylacetic acid (3-HPAA) was administered as a single bolus, whereas in another set, it was given as four subsequent 5 min lasting infusions; (4) arterial blood pressure and heart rate were recorded (see Section 2 for details). The second panel (**b**) depicts the ex vivo experiments performed: (1) the left circumflex porcine coronary artery was isolated and cut into small arterial rings; (2) the rings were placed into an isolated tissue bath of modular type and kept in the Krebs solution at 37 °C, under adequate oxygenation (carbogen 95% O_2_/5% CO_2_); (3) the rings were precontracted with KCl and the vasodilatory activity of 3-HPAA was confirmed by measuring changes in isometric tension developed by 3-HPAA (0.1, 0.5 and 1 mM); (4) in a separate set of experiments, the mechanism of action of 3-HPAA was explored by using intact endothelium and denuded rings and a series of inhibitors/antagonists/activators (L-NAME, TRAM-34, UCL-1684, indomethacin, atropine, Bay K8644).

Twelve SHR rats (average weight 356 ± 21 g, blood pressure under anesthesia 211 ± 64/152 ± 45 mmHg, heart rate 381 ± 37 bpm) were anesthetized i.p. by pentobarbital (50 mg·kg^−1^). The pressure transducer MLT0380/D was linked to the left common carotid artery, and arterial blood pressure with heart rate were recorded using a Power Lab device connected to the LabChart 7 software (ADInstruments, Sydney, Australia). The left saphenous vein was cannulated for i.v. administration of a bolus or an infusion. After surgery, each rat was allowed to stabilize for 15 min before the experiment. 3-HPAA was dissolved in saline and administered under two protocols: (1) as a single bolus dose ranging from 0.001 to 10 mg·kg^−1^ (the volume applied was always 0.1 mL, *n* = 8); or (2) as four subsequent 5 min-lasting infusions at doses 0.05, 0.25, 1 and 5 mg·kg^−1^·min^−1^ (*n* = 4) in the infusion rate of 50 μL per minute using the “Genie” Kent syringe pump (Kent Scientific Corporation, Torrington, CT, USA) (Figure 2a). In both protocols, the next dose was always given after blood pressure was stable for at least 5 min. As a control, each animal was treated with saline under the same conditions before the first administration of 3-HPAA was carried out. After the measurement, each rat was sacrificed by i.v. KCl (1 mL, 1 mol·L^−1^). The body temperature during the experiment was maintained at 36.5 ± 0.5 °C using a heating plate.

### 2.4. Ex Vivo Experiments

#### 2.4.1. Tissue Preparation

The ex vivo experiments were performed on porcine coronary arterial ring segments (Figure 2b). Porcine hearts (*n* = 11) were obtained from a local slaughterhouse. Pigs were healthy animals of both sexes, from standard breeding and under veterinary control, intended for consumption. The hearts were processed for the experiment approximately 4 h after the slaughter of the animal. The hearts were immersed in the Krebs solution, and the left circumflex coronary artery was immediately excised, again immersed in the Krebs solution and cleaned of adherent fat, connective tissue and blood. Afterwards, the purified coronary artery was cut into cylindrical rings of approximate 3 mm length. For certain mechanistic experiments, the endothelial layer was mechanically disrupted by gently rubbing the luminal surface with forceps. The rings were maintained in tissue baths filled with the Krebs solution under oxygenated conditions (95% O_2_ and 5% CO_2_) at 37 °C. Each artery ring was hung between two stainless-steel wire hooks, one of them fixed to a holder and the second connected to a transducer and computer equipped with S.P.E.L. Advanced Kymograph Software, v3.2 (Experimetria Ltd., Budapest, Hungary). This arrangement enables the measurement of isometric tension. The preparations were equilibrated at a tension of 2 g for 40 min, during which the Krebs solution was replaced every 10 min. After that, the tension was set at 1 g (baseline), each tissue bath was filled with 5 mL of the Krebs solution, and the following viability test was performed: the coronary rings were contracted with KCl (final concentration inside the bath was 40 mM), and when the plateau was attained, bradykinin (300 nM) was added to confirm intact endothelium or denuded rings. Then, the coronary rings were washed several times with the Krebs solution. 

#### 2.4.2. Confirmation of the Vasodilatory Activity of 3-HPAA on the Porcine Coronary Artery

The vasorelaxant activity was measured on KCl-precontracted (40 mM) arterial rings (Figure 2b). Once plateau had been reached, 3-HPAA was cumulatively added to the bath in concentrations ranging from 100 µM to 1 mM. 3-HPAA was prepared in the Krebs solution. The Krebs solution without the tested compound was added to some coronary rings as the control using the same procedure. Each dose was applied after stabilization of the relaxant response caused by the previous dose, and sodium nitroprusside (100 μM) was used to induce maximal relaxation in all experiments at the end of the procedure. 

#### 2.4.3. Mechanistic Experiments

In the following experiments, the mechanism of vasodilatory action of 3-HPAA was studied on intact endothelium coronary rings using a nitric oxide (NO) synthase inhibitor, L-NAME (100 μM); a muscarinic receptor antagonist, atropine (50 μM); a cyclooxygenase (COX) inhibitor, indomethacin (10 μM); or the intermediate and small conductance Ca^2+^-activated K^+^ channels inhibitors, TRAM-34 (10 μM) and UCL-1684 (1 μM), respectively (Figure 2b). Atropine or inhibitors were always added 30 min before the addition of KCl (40 mM). Afterwards, in a separate experiment, the involvement of vascular Ca_V_1.2 Ca^2+^ channels (L-type) on 3-HPAA-induced vasorelaxation was investigated by pre-treating endothelium-denuded coronary rings with 15 mM KCl followed by incubation with the 3-HPAA at a concentration of 1 mM for 30 min. Then, an L-type calcium channel activator, Bay K8644, was applied cumulatively (100 pM–10 µM), in the dark, to induce a contractile response. KCl (80 mM) was used to induce maximal contraction. Rings incubated with the corresponding volume of the vehicle (the Krebs solution) were used as negative controls. In addition, rings incubated with the known L-type calcium channel inhibitor nifedipine (150 nM) were used as positive controls. The investigated target proteins and the ligands used in the mechanistic experiments are summarized in Table 1.

### 2.5. Data Analysis

GraphPad Prism 7.03 (GraphPad Software, San Diego, CA, USA) was used for data analysis. For in vivo experiments, differences between individual bolus doses of 3-HPAA and control (saline) were compared using a one-way ANOVA, followed by the Dunnett post hoc test. For infusions, a two-way ANOVA followed by the Dunnett post hoc test was employed. For the mechanistic ex vivo experiments, the results were compared by using an unpaired *t*-test. For experiments with Bay K8644, two-way ANOVA was used followed by a Dunnett test for multiple comparisons. Data are shown as mean ± SEM.

## 3. Results

### 3.1. Effect of 3-HPAA on the Blood Pressure and Heart Rate In Vivo in SHR

#### 3.1.1. Single Bolus Administration

**Figure 3:**
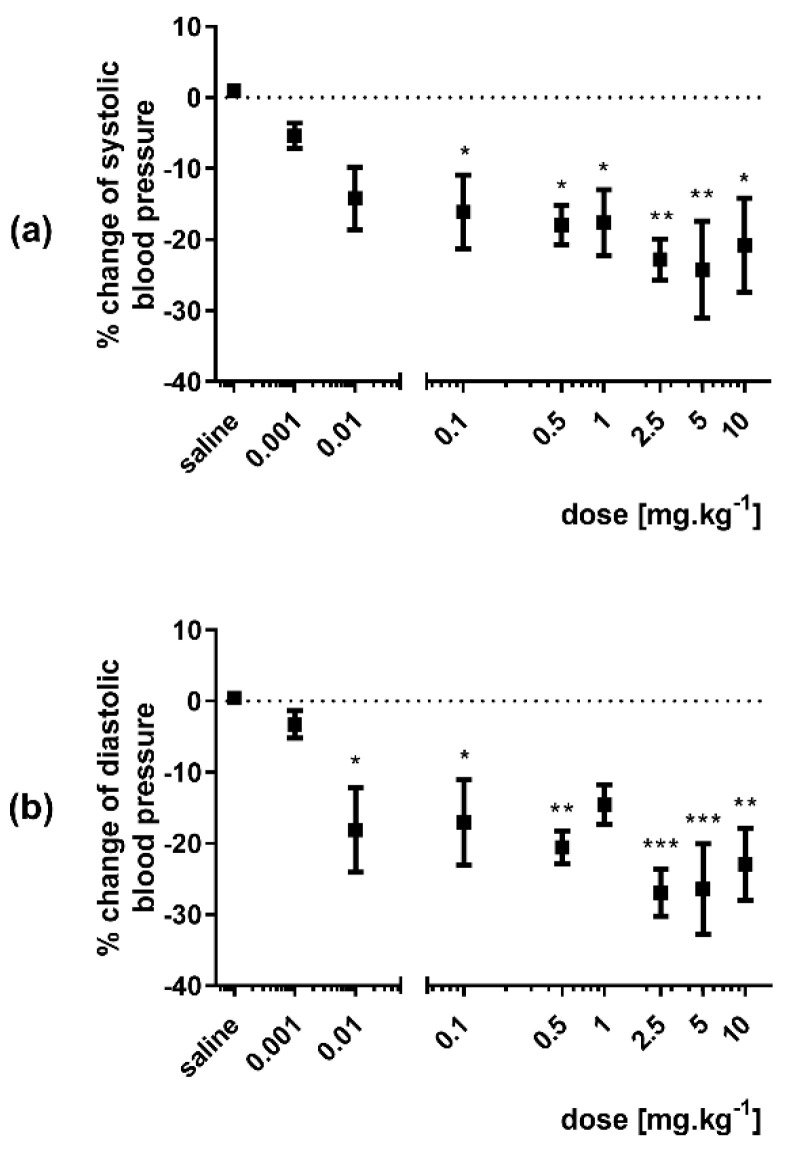
Changes in arterial systolic (**a**) and diastolic (**b**) blood pressures after bolus i.v. administration of 3-hydroxyphenylacetic acid (doses ranging from 0.001 to 10 mg·kg^−1^) expressed as percent change from the initial value in spontaneously hypertensive rats (*n* = 8). Significant change: * *p* < 0.05, ** *p* < 0.01, *** *p* < 0.001 vs. saline.

A significant decrease in mean, systolic and diastolic blood pressures was observed after bolus administration of 3-HPAA in doses ranging from 0.01 to 10 mg·kg^−1^ (Figure 3 and Figure S1). The decrease in systolic blood pressure was apparent from a dose of 0.1 mg·kg^−1^ (Figure 3a), whereas diastolic blood pressure dropped significantly even from a 10 times lower dose (0.01 mg·kg^−1^) (Figure 3b). In the same experiment, no effect on heart rate was found at any of the 3-HPAA doses (Figure S2).

#### 3.1.2. Infusion Administration 

**Figure 4:**
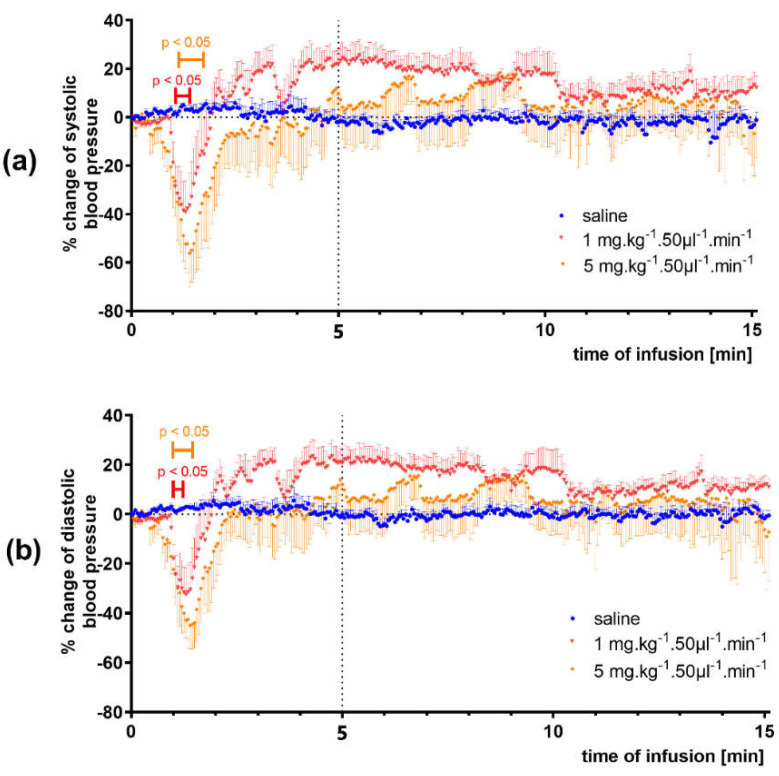
Effect of 3-hydroxyphenylacetic acid (1 and 5 mg·kg^−1^·min^−1^) infusions on systolic blood pressure (**a**) and diastolic blood pressure (**b**) in spontaneously hypertensive rats (*n* = 4). Significant change: *p* < 0.05.

The 5 min-lasting infusions of 3-HPAA resulted in a decrease in mean, systolic and diastolic blood pressures (complete results in Figure S3). The effect was dose-dependent, reaching maximally approx. 50%. Significant changes were produced by the two highest doses, 1 and 5 mg·kg^−1^·min^−1^ (Figure 4). Additionally, in this case, no significant changes in heart rate were found (Figure S4).

### 3.2. Confirmation of the Vasodilatory Properties of 3-HPAA on Porcine Coronary Artery

**Figure 5:**
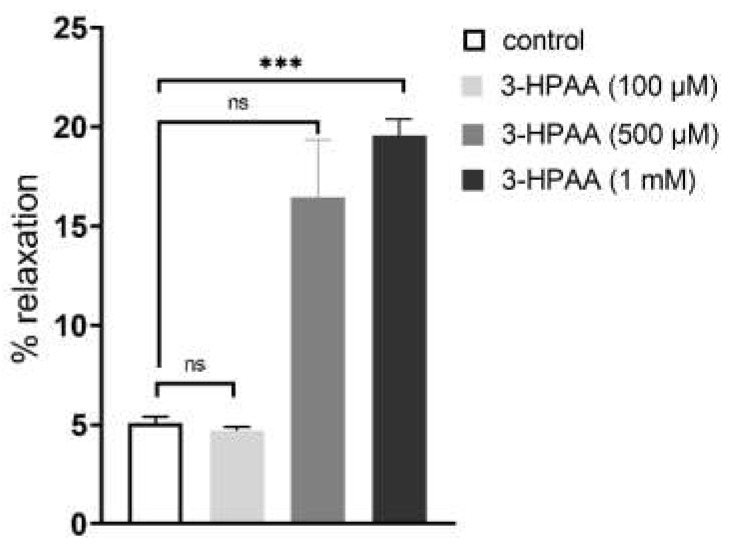
Confirmation of the vasodilatory effects of 3-hydroxyphenylacetic acid (3-HPAA) in porcine coronary arterial rings (*n* = 6). Data are expressed as % from the maximal relaxation induced by sodium nitroprusside (100 µM). Significant change: *** *p* < 0.001 vs. control, ns—not significant.

In ex vivo experiments, 3-HPAA induced vasodilation of pig coronary artery in a dose-dependent manner (Figure 5). Except for the first dose, vasodilation was observed. The results were, however, significant only for the highest concentration (1 mM). The same concentration was used for further mechanistic testing ex vivo.

### 3.3. Mechanism of the Vascular Effects of 3-HPAA Studied Ex Vivo on Porcine Coronary Artery 

**Figure 6:**
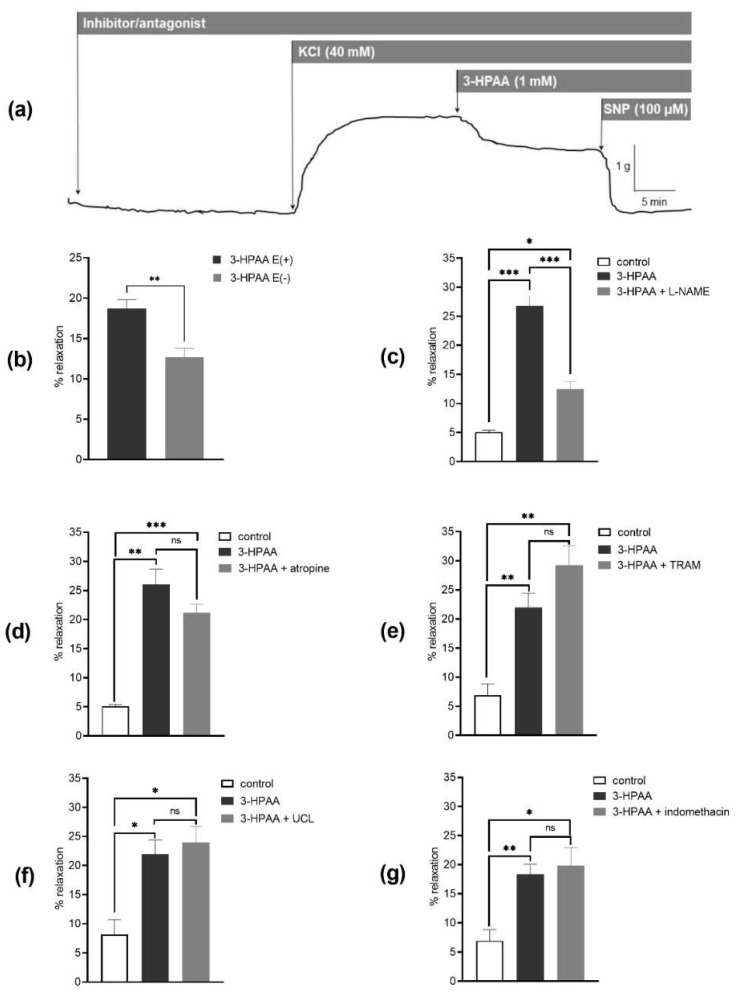
Impact of endothelium and different inhibitors/antagonists on the vasodilatory effect of 3-hydroxyphenylacetic acid (3-HPAA). Scheme of the representative isometric tension traces of the porcine coronary arterial rings (**a**). Ex vivo vasodilatory effects of 3-HPAA (1 mM) were evaluated on endothelium-intact E(+) and endothelium-denuded E(−) porcine coronary arterial rings precontracted with KCl (40 mM) (**b**). In another experiment set, intact coronary rings were used and the percentage of relaxation was evaluated in the presence of: NO-synthase inhibitor L-NAME (100 µM) (**c**), or the M-receptor antagonist atropine (50 µM) (**d**), or Ca^2+^-activated K^+^ channel inhibitors, TRAM-34 (10 µM) (**e**), or UCL-1684 (1 µM) (**f**), or the cyclooxygenase inhibitor indomethacin (10 µM) (**g**). Data were calculated from the maximal relaxation induced by sodium nitroprusside (100 µM). Significant change: * *p* < 0.05, ** *p* < 0.01, *** *p* < 0.001 (*n* = 6, for control *n* = 4), ns—not significant.

**Figure 7:**
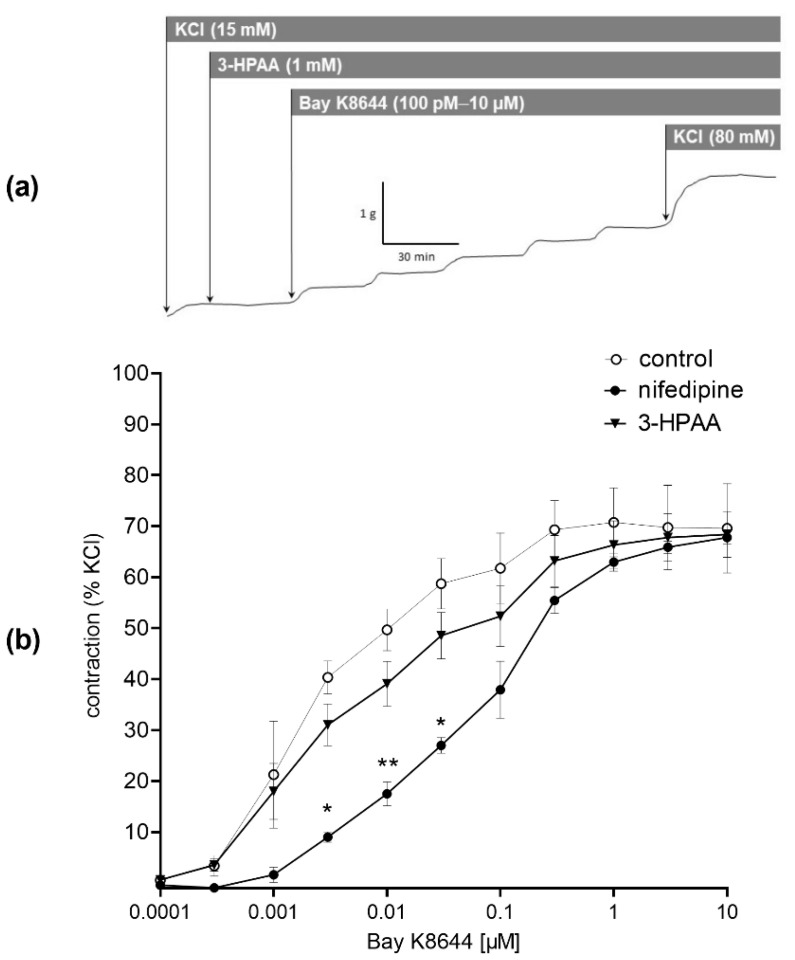
Scheme of the representative isometric tension traces of the porcine coronary arterial rings (**a**). Effect of pre-treatment of coronary rings with 3-hydroxyphenylacetic acid (3-HPAA, 1 mM) on contraction induced by cumulative doses of the L-type calcium channel activator, Bay K8644 (100 pM–10 µM). Nifedipine (150 nM) and vehicle (the Krebs solution) were used as positive and negative controls, respectively (**b**). Data were calculated from the maximal contraction induced by KCl (80 mM). Significant change: * *p* < 0.05, ** *p* < 0.01 vs. the control (*n* = 7 for 3-HPAA, for both controls *n* = 4).

To investigate the mechanisms underlying the 3-HPAA-induced vasodilation, we compared its effects on endothelium-intact and endothelium-denuded porcine coronary arterial rings precontracted with KCl. The endothelium removal decreased the vasodilatory effect (Figure 6b). Following on from this finding, the involvement of endothelial NO was tested. Pre-treatment with an inhibitor of eNOS, L-NAME (100 µM), also resulted in a significant decrease in 3-HPAA-induced vasodilation (Figure 6c). In contrast, analogous pre-treatment of coronary rings with muscarinic receptor antagonist atropine (50 µM), or with IKCa or SKCa channel blockers, TRAM-34 (10 µM) and UCL-1684 (1 µM), respectively, or with the COX inhibitor, indomethacin (10 µM) did not cause any changes in vasodilation produced by 3-HPAA (Figure 6d–g). Finally, we decided to investigate whether 3-HPAA may act through inhibition of Ca_V_1.2 calcium channels (L-type) on vascular smooth muscle cells. Pre-treatment of the rings with 3-HPAA (1 mM) had no significant effect on contraction induced by cumulative doses of Bay K8644 (100 pM–10 µM) (Figure 7), while incubation with the known Ca_V_1.2 calcium channel (L-type) inhibitor nifedipine (150 nM) significantly reduced the contractions (positive control). 

## 4. Discussion

Screening of different phenolic metabolites of flavonoids showed that 4 of 22 were potent vasodilators. Following on from these results, the cardiovascular effects of 3-(3-hydroxyphenyl)propionic acid, 3,4-dihydroxyphenylacetic acid (DHPA) and 4-methylcatechol were confirmed in vivo, and possible mechanisms of their actions were studied ex vivo [14,15]. Another flavonoid metabolite, 3-HPAA, was temporarily excluded from further investigation since it was not able to produce full relaxation in the initial screening on the rat aorta. In this work, we decided to extend our previous studies and focus on 3-HPAA as well. We aimed to (1) confirm its vasodilatory effect both in vivo in spontaneously hypertensive rats and ex vivo in another experimental model (porcine coronary arterial rings), and (2) study the mechanism of this action.

**Effect of 3-HPAA on the Blood Pressure and Heart Rate In Vivo in SHR**

While 3-HPAA was a less potent vasodilator in the previous ex vivo screening [14], within this work, it was clearly confirmed that is able to decrease blood pressure in vivo in spontaneously hypertensive rats after intravenous administration. When administered as a bolus, the mean, systolic and diastolic blood pressures decreased. Quite unexpectedly, a significant effect on diastolic blood pressure was already observed after a very low dose of 10 μg·kg^−1^. The extent of this response was relatively high (approx. 20%). With increasing doses of 3-HPAA, the effect increased only slightly to approx. 25%. The same was true in the case of systolic blood pressure, when a significant decrease (approx. 15%) was present after the dose of 100 μg·kg^−1^, and the maximal decrease found was approx. 25%. There was variability among animals resulting in variable values; however, the dose-dependency of the effect was obvious. In contrast, no changes in heart rate were observed after intravenous bolus application. This can mean that the heart was not involved in the blood pressure-decreasing effect and the effect was solely based on peripheral relaxation. Absence of heart rate changes is important also from a safety point of view since the feedback increase in heart rate caused by the sympathetic nervous system, which follows a pronounced decrease in arterial blood pressure, is clinically unsuitable, as was well reported with the calcium channel blocker nifedipine [24].

In real situations, the colonic metabolites of ingested flavonoids are continuously absorbed from the GIT. To mimic this, slow intravenous infusions of 3-HPAA at different rates were administered in another set of experiments. Analogously to previous bolus application, a decrease in mean, systolic and diastolic blood pressures was observed. The effect was dose dependent, and a significant effect was brought about by the doses of 1 and 5 mg·kg^−1^·min^−1^ reaching maximally approx. 50% drop in blood pressure. Again, no significant changes in heart rate were observed during the 5 min-lasting-infusion, nor during the next 10 min of animal monitoring. 

This effect on blood pressure can have a real impact. Flavonoids from the diet are rather poorly absorbed in the small intestine. They reach the colon and undergo microbial metabolism. The bacterial degradation process consists of a reduction of the double bond in the 2,3-position followed by the C-ring fission. The next step depends on the presence or absence of the 3-hydroxyl group. Flavones that do not possess this hydroxyl give origin to hydroxyphenylpropionic acid derivatives, while flavonols with the 3-hydroxyl give origin to derivatives of hydroxyphenylacetic acids [25]. However, 3-HPAA arises as a ring-fission product derived from microbial catabolism of many parent flavonoids, not only flavonols such as quercetin. Moreover, an important metabolite with vasodilatory activity, DHPA, gives origin to 3-HPAA after its dehydroxylation and to a smaller extent to another vasorelaxant compound, 3,4-dihydroxybenzoic acid/protocatechuic acid. 3-HPAA is further catabolized into vaso-inactive hippuric and benzoic acids (Figure 1) [15,26].

In general, small flavonoid metabolites show higher plasma levels than their parent flavonoids and can reach peak concentrations usually in the range of 1 to 615 nM or even 42.9 µM in some cases [17]. The same seems to be true for 3-HPAA. In animal studies, administration of calafate berries extract (providing ~2.6 mg of phenolics) through gavage to gerbils resulted in maximal plasma concentrations of 3-HPAA of approx. 300 nM after 4 h [27]. Other authors reported that a single 3-HPAA intravenous bolus of 2 and 4 mg·kg^−1^ to rats resulted in maximal plasma concentrations of approx. 6 mg·L^−1^ (~40 µM) and 16 mg·L^−1^ (~100 µM) [28]. In our study, the maximal dose administered intravenously as a bolus was 10 mg·kg^−1^. Thus, we can estimate [28] that the maximal plasma level achieved could be between 100 and 200 µM, which is 11–22 times higher than was physiologically detected [17]. Importantly, significant effects on the diastolic blood pressure were observed already at the dose of 10 μg·kg^−1^, which could roughly correspond to an achievable concentration of 100–200 nM. Analogous is true for administration via infusion. A dose of 1 mg·kg^−1^·min^−1^ could result in plasma concentration of roughly 10 µM [28], which is within the range of total levels produced by diet [17]. In contrast, the dose of 5 mg·kg^−1^·min^−1^ would be difficult to achieve through a diet rich in polyphenols, and in this case, 3-HPAA may be, instead, applicable as a drug or supplement. Extrapolation of the animal data to humans is not easy; however, these concentrations are achievable in humans after consumption of a diet rich in flavonoids and might be associated with the effect on the vascular system [17]. Unfortunately, the kinetic data on 3-HPAA are still limited. An 8-week placebo-controlled dietary trial with 72 participants showed a significant increase in plasma levels of 3-HPAA (from ~180 to ~250 nM) after consumption of berries, which provided about 837 mg of polyphenols per day. Moreover, there was also an increase of 87% in the urinary excretion of 3-HPAA [29]. Another study detected 60 different phenolic metabolites in plasma and urine in 10 volunteers after consumption of cranberry juice containing 787 mg of polyphenols. 3-HPAA was among the metabolites determined in plasma and reached a maximum concentration of ~600 nM after 10 h approximately [17]. Another kinetic study with nine healthy young men showed that the bioavailability of (poly)phenols does not depend solely on the amount taken. The amount of 766 mg of polyphenols led to maximum 3-HPAA plasma concentrations of ~260 nM, whereas ingestion of more than double the amount gave rise to even lower levels (~240 nM) [30]. Nevertheless, in both cases, the plasma concentrations reached roughly 250 nM. 

**Mechanism of the Vascular Effects of 3-HPAA Studied Ex Vivo**

We performed data search in the PubMed database with the keyword “3-hydroxyphenylacetic acid”. Analysis of the 110 articles found allowed us to conclude that data on 3-HPAA pharmacokinetics are limited and pharmacodynamic studies, to our best knowledge, do not exist. Only one study showed that 3-HPAA decreases COX-2 protein levels in colon cancer cells, but without an effect on PGE_2_ production [31]. 

As in our in vivo experiments, 3-HPAA decreased blood pressure dose dependently and had no impact on heart rate, we hypothesized that the mechanism of the observed effects could lie in the direct action of 3-HPAA on the vasculature. Therefore, we performed various complementary ex vivo experiments to explore the mechanism of action. As there is an apparent appeal in the Czech Republic to decrease the use of laboratory animals in line with the 3Rs (Replacement, Reduction and Refinement), we selected an alternative model consisting of the use of porcine coronary arteries from fresh hearts, which were obtained from a local slaughterhouse. This model was not optimal. First, vasodilation was observed at a much higher concentration than in rat aorta, and second, this concentration is roughly 100 times higher than the concentration achievable through diet [17]. Notwithstanding this limitation, this setting allowed us to see differences between experimental groups and, hence, it served sufficiently for the determination of the mechanism of action. On the other hand, the advantage was that pigs and humans apparently share numerous similarities concerning the cardiovascular system [32]. It was shown that 3-HPAA produces a dose-dependent vasodilation of pig coronary arteries ex vivo. This effect was at least partially mediated by endothelium with participation of the endothelium-derived NO. In contrast to NO, we did not confirm the participation of endothelial M receptors, COX, SK_Ca_ and IK_Ca_ channels, nor direct effects on smooth muscle Ca_V_1.2 channels (L-type). 

**Figure 8:**
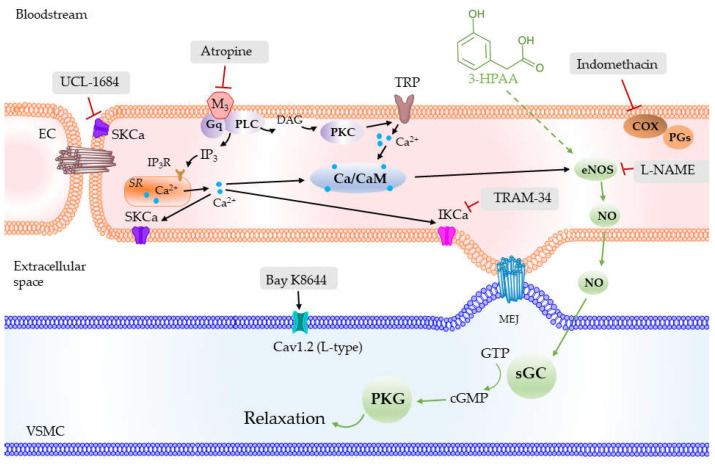
Schematic depiction of the possible mechanism of action of 3-hydroxyphenylacetic acid (3-HPAA) involving the production of NO in endothelial cells and activation of sGC in vascular smooth muscle cells (green arrows) and other pathways investigated in the current study. SKCa—small conductance Ca^2+^-activated K^+^ channels; IKCa—intermediate conductance Ca^2+^-activated K^+^ channels; M_3_—muscarinic receptor subtype M_3_; PLC—phospholipase C; DAG—diacylglycerol; PKC—protein kinase C; IP_3_—inositol trisphosphate; IP_3_R—inositol trisphosphate receptor; SR—sarco/endoplasmic reticulum; TRP—transient receptor potential channel; Ca/CaM—calcium-calmodulin complex; COX—cyclooxygenase; PGs—prostaglandins; eNOS—endothelial nitric oxide synthase; NO—nitric oxide; MEJ—myoendothelial junction; sGC—soluble guanylate cyclase; GTP—guanosine triphosphate; cGMP—cyclic guanosine monophosphate; PKG—protein kinase G; Cav1.2 (L-type)—L-type calcium channels; EC—endothelial cell; VSMC—vascular smooth muscle cell.

NO was previously referred to as endothelium-derived relaxing factor and its role in the vascular physiology is well known. Briefly, after being synthesized by eNOS in endothelial cells, NO diffuses to vascular smooth muscle cells where it activates soluble guanylate cyclase (sGC) and the cGMP-PKG pathway (Figure 8). PKG, in turn, activates various K^+^ channels present on the smooth muscle, namely large-conductance calcium-activated (BK_Ca_), ATP-sensitive (K_ATP_), inward rectifier (K_IR_) and voltage-gated (K_V_), thus allowing the transfer of K^+^ ions. This leads to an increase in negative membrane potential with the consequent inhibition of voltage-gated calcium channels (mainly L-type) and blockade of extracellular Ca^2+^ influx. The levels of intracellular Ca^2+^ are also regulated through activation of SERCA either by PKG or directly by NO [33], and by inhibition of IP_3_R channels. The lack of NO-mediated effects is related to various pathologies. A decreased expression and activity of eNOS was found in aortas from SHR [34], and an impaired NO production was demonstrated in endothelial and vascular smooth muscle cells from mesenteric arteries and aorta of genetically modified hypertensive rats [35]. In human studies, an abnormal endothelial function was reported in patients with essential hypertension [36]. Endothelial dysfunction has been associated with impaired vascular bioavailability of NO [37] without specifying whether this mechanism is based on a reduction in synthesis, release, or diffusion of NO. Recently, the mechanisms postulated also include an increase in oxidant status that promotes NO breakdown [38,39]. Thus, the mechanisms described above might contribute to the vasodilatory effect of 3-HPAA (Figure 8), and its potential protective vascular effects. Importantly, this can be the reason for the large differences between a very high sensitivity of SHR to 3-HPAA vasodilatory effects and the low sensitivity of healthy coronary vessel from pig hearts, although this theory must be confirmed in additional studies.

If this is true, the question of 3-HPAA-induced NO synthesis arises. The pKa value of 3-HPAA is 4, which means that under physiological pH, the substance is mostly ionized. This hinders passive transmembrane transport; however, the presence of a transporting system was not verified and cannot be excluded. The activation of eNOS is often triggered by an increase in cytosolic Ca^2+^. In our experiments, 3-HPAA vasodilation was not dependent on the activity of the endothelial IKCa and SKCa channels. As both these channels are Ca^2+^-sensitive [40], the previous increase in cytoplasmatic Ca^2+^ levels is not probable. Accordingly, endothelial M-receptors, which are GPCRs coupled to the second messengers’ DAG+IP_3_/PKC+ cytosolic Ca^2+^ increase, were not involved, as their blockade by atropine did not modify the vasodilation caused by 3-HPAA. This observation, together with the fact that no changes occurred in the heart rate of rats, suggest that a direct cholinomimetic activity for 3-HPAA is unlikely. Of note, there is a large homology in the muscarinic receptors described among mammals: M_1_ to M_5_ in rats, and M_1_ to M_3_ in pigs, have revealed a homology greater than 90% with the human amino acid sequences of these receptors [41]. Last but not least, in addition to NO, endothelial vasodilation can also be mediated by other endothelial products, among them a mediator from cyclooxygenase-pathway prostacyclin (PGI_2_). This is not probable in the case of 3-HPAA because the presence of indomethacin, a cyclooxygenase blocker, had no effect. 

Previously, we studied another important colonic metabolite, DHPA, on the rat aorta ex vivo. Its vasodilatory effect was also partially dependent on endothelium, but with the involvement of endothelial IK_Ca_ channels and COX, thus Ca^2+^ being dependent [15]. This would imply that vasodilatory colonic metabolites of flavonoids may act through different mechanisms of action. In real conditions, the presence of different mechanisms and the interplay of several colonic metabolites might facilitate vasodilation. This hypothesis is in accordance with our in vivo study on mixtures of colonic metabolites [42]. Interestingly, some studies evidenced that the parent flavonoid, quercetin, is also vasoactive. It activates eNOS, and this action is mediated by an increase in cytosolic Ca^2+^_,_ activating thereafter the Ca^2+^-activated K^+^ channels, mainly SK_Ca_, and causing hyperpolarization of endothelial cells [43,44]. Quercetin, hence, acts in a different way. However, the bioavailability of parent quercetin is low [45,46] and, thus, its direct impact on vasodilation is probably not crucial.

The research published in this paper has several limitations. For instance, the porcine coronary artery is not a resistance vessel, while the arterial blood pressure-decreasing effect is likely associated with the dilation of resistance vessels. An i.v. administration of a single bolus or even slow i.v. infusion does not mimic a real exposure scenario in which humans ingest multiple doses of flavonoids, mainly in the form of glycosides, throughout the day. Thus, the plasma profile of metabolites may differ. Importantly, the flavonoids ingested are metabolized not into one but into a mixture of metabolites. Many of them may be vasoactive and the interplay among them influences the final effect. Furthermore, i.v. application does not allow for assessment of the role of the intestinal microbiota and variability in the production of 3-HPAA and other metabolites from parent flavonoids. Future studies should try to address these issues to better understand the bioactivities and the mechanism of action of 3-HPAA in the cardiovascular system, including the possible role of NO.

## 5. Conclusions

The data provided strong evidence that the flavonoid metabolite, 3-HPAA, formed by the human gut microbiota, is vasoactive and decreases blood pressure. In addition, the results suggest that a decrease in blood pressure can be attained at achievable concentrations. Along with these findings, we demonstrated that the hypotensive effect was not the result of a direct action on the heart but was more likely vascular based. Finally, the 3-HPAA-induced vasodilation was, at least partially, mediated by endothelium, where NO-dependent effects might play a role. 

## Figures and Tables

**Table 1:** Target proteins investigated in the 3-hydroxyphenylacetic acid(3-HPAA) mechanistic studies and the ligands used in the ex vivo experiments on the porcine coronary artery.

Targets	Ligands
**Ion channels**	
SKCa	UCL-1684
IKCa	TRAM-34
L-type Ca^2+^ channels (Cav1.2)	Bay K8644
	nifedipine
**Enzymes**	
eNOS	L-NAME
cyclooxygenase	indomethacin
**GPCRs**	
M receptors	atropine

SKCa, small-conductance calcium-activated potassium channels; IKCa, intermediate-conductance calcium-activated potassium channels; eNOS, endothelial nitric oxide synthase; GPCRs, G protein-coupled receptors; M receptors, muscarinic receptors; L-NAME, L-Nω-nitro arginine methyl ester. The subgroups of the targets are in bold.

## Supplementary Materials

The following supporting information can be downloaded at: www.mdpi.com/article/10.3390/nu14020328/s1. Figure S1. Changes in mean blood pressure after bolus i.v. administration of 3-HPAA. Figure S2. Changes in heart rate after bolus i.v. administration of 3-HPAA. Figure S3. Effect of 3-HPAA infusions (0.05, 0.25, 1 and 5 mg·kg^−1^·min^−1^) on systolic, diastolic and mean blood pressures in spontaneously hypertensive rats. Figure S4. Effect of 3-HPAA infusions (0.05, 0.25, 1 and 5 mg·kg^−1^·min^−1^) on heart rate in spontaneously hypertensive rats.
