# Supplementary material for: 3-Hydroxyphenylacetic Acid: A Blood Pressure-Reducing Flavonoid Metabolite
Source: Nutrients. 2022 Jan 13;14(2):328. doi: 10.3390/nu14020328 (PMC8781193; doi:10.3390/nu14020328)
Supplement: Supplementary file 1 [file nutrients-14-00328-s001.zip › nutrients-1533221-supplementary.pdf]

nutrients

3-hydroxyphenylacetic acid: a blood pressure-reducing flavonoid metabolite

Patrícia Dias, Jana Pourová, Marie Vopršalová, Iveta Nejmanová & Přemysl Mladěnka

Charles University, Faculty of Pharmacy, Czech Republic

Supplementary data

4 pages

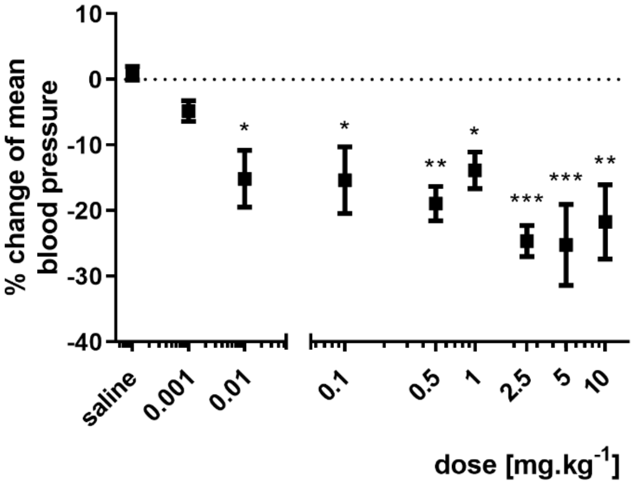

Figure S1. Changes in mean arterial blood pressure after *bolus* i.v. administration of 3-hydroxyphenylacetic acid. Doses ranged from 0.001 to 10 mg·kg^{-1}. Results are expressed as per cent decrease from the initial value. Spontaneously hypertensive rats (n=8) were used. The decrease in mean blood pressure was significant from a low dose of 0.01 mg·kg^{-1}. * $p < 0.05$, ** $p < 0.01$, *** $p < 0.001$ *vs.* saline.

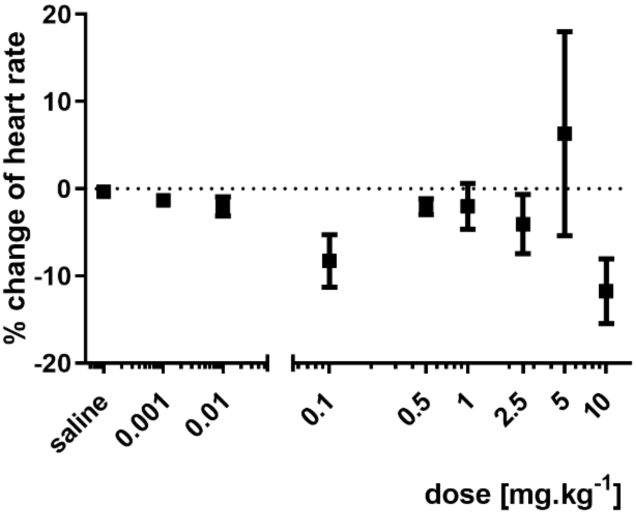

Figure S2. Changes in heart rate after *bolus* i.v. administration of 3-hydroxyphenylacetic acid. Doses ranged from 0.001 to 10 mg·kg^{-1}. Results are expressed as per cent change from the initial value in spontaneously hypertensive rats (n=8). No significant differences were found.

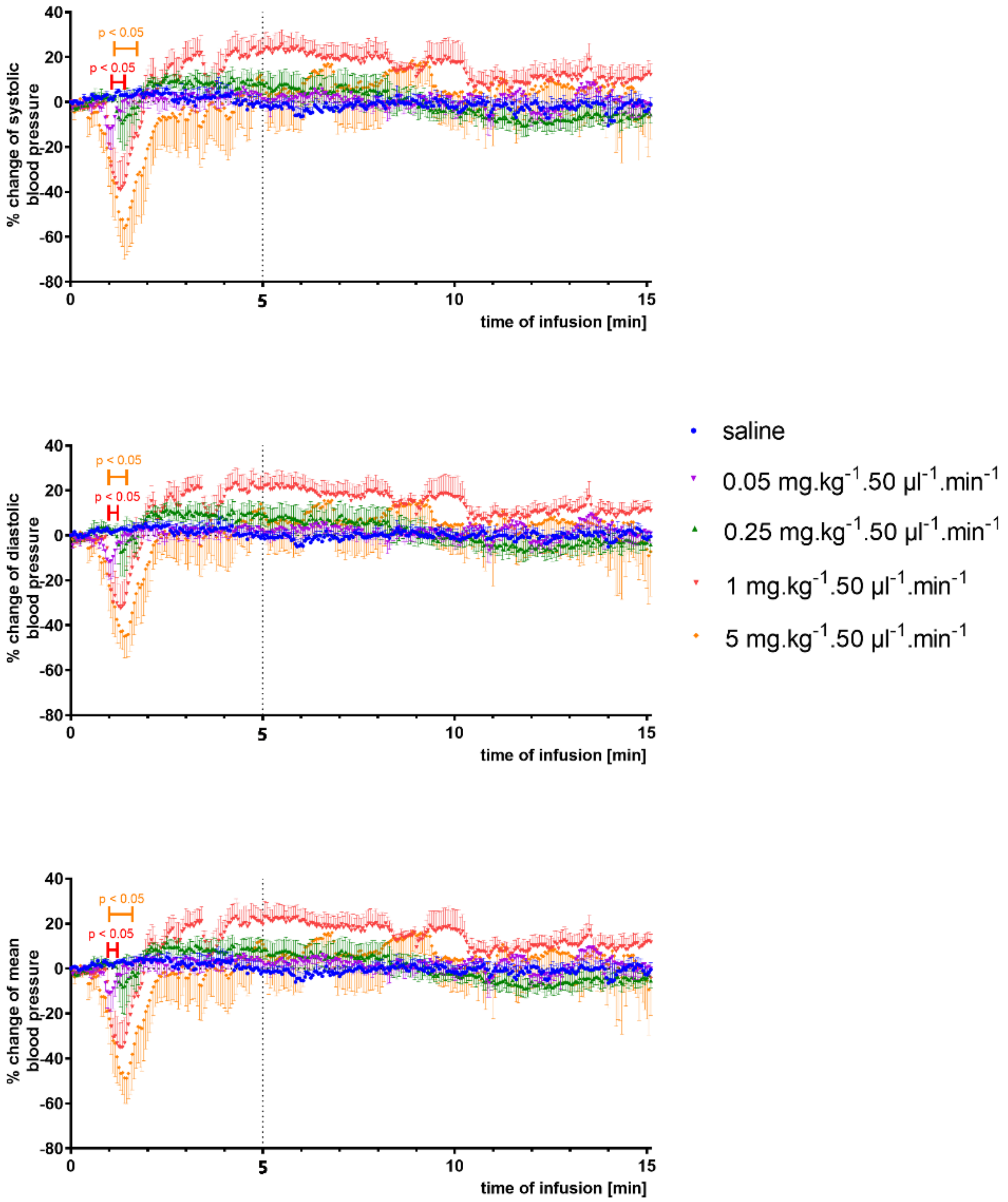

Figure S3. Effect of 3-hydroxyphenylacetic acid infusions (0.05, 0.25, 1 and 5 mg·kg^{-1}·min^{-1}) on systolic, diastolic and mean blood pressures in spontaneously hypertensive rats.

Saline was used as the solvent and hence also as the control. $p < 0.05$ *vs.* saline (n=4).

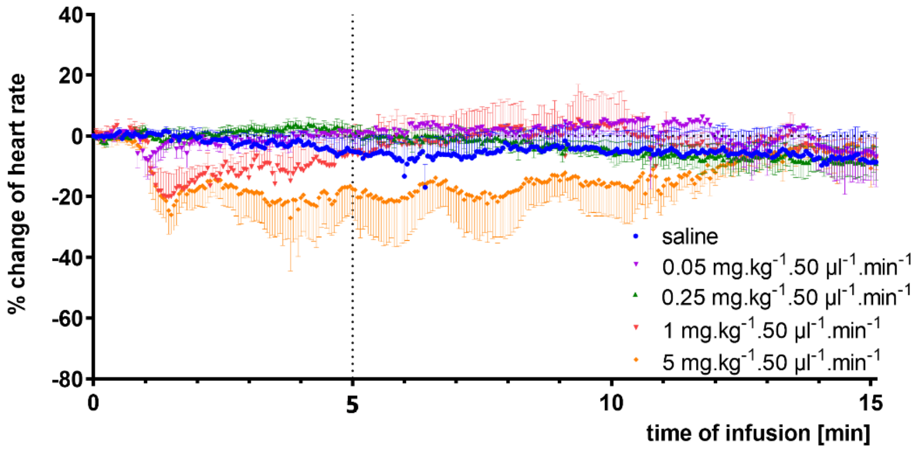

Figure S4. Effect of 3-hydroxyphenylacetic acid infusions (0.05, 0.25, 1 and 5 mg·kg^{-1}·min^{-1}) on heart rate in spontaneously hypertensive rats.

No significant differences were found (n=4).
